# Draft Whole-Genome Sequences of 10 Serogroup O6 Enterotoxigenic *Escherichia coli* Strains

**DOI:** 10.1128/genomeA.01274-14

**Published:** 2014-12-18

**Authors:** Vaishnavi Pattabiraman, Cheryl A. Bopp

**Affiliations:** Centers for Disease Control and Prevention, Atlanta, Georgia, USA

## Abstract

Entertotoxigenic *Escherichia coli* (ETEC) is a major cause of global diarrhea, resulting in approximately 200 million occurrences and 300,000 to 400,000 deaths annually, primarily in children under the age of five. Here, we announce the release of the draft genomes of 10 ETEC isolates belonging to serogroup O6.

## GENOME ANNOUNCEMENT

Enterotoxigenic *Escherichia coli* (ETEC) is an enteric pathogen that causes traveler’s diarrhea and is a leading cause of infectious diarrhea in children under the age of five in developing nations. ETEC is one of the four enteric pathogens that causes more than one-half of all diarrheal deaths in the world and by itself causes ~200 million cases of watery diarrhea and about 300,000 to 400,000 diarrheal deaths annually in children under the age of five (1). The heat-labile enterotoxin (LT), heat-stable enterotoxins 1a (ST1a), and ST1b are classical ETEC virulence genes that induce water and electrolyte loss from the infected intestines, leading to diarrhea (2–4). The complete genomes of four human ETEC strains, *E. coli* H10407 (5), E24377A (6), B2C (7), and W25K (8), have been published. In this announcement, we report the first draft whole-genome sequences of 10 serogroup O6 ETEC strains from historical and recent outbreaks (Table 1).

**TABLE 1:** Characteristics of the 10 genomes of ETEC isolates

ETEC isolate	Serotype	ETEC virulence genes	NCBI accession no.	No. of contigs	Genome size (bp)	No. of coding sequences	Country/location of outbreak
2013EL-1319^*a*^	O6:H16	*st1b, prfB*	JPIF00000000	189	4,712,597	4,327	Cruise ship
2013EL-1320^*a*^	O6:H16	*st1b, prfB*	JPUD00000000	297	4,711,514	4,369	Cruise ship
2011EL-1369-1^*b*^	O6:H16	*eltA, st1b, prfB*	JPUS00000000	324	4,801,047	4,410	United States
2011EL-1370-2^*b*^	O6:H16	*eltA, st1b, prfB*	JPUT00000000	287	4,808,745	4,402	United States
M9803	O6:H16	*eltA, st1b, prfB*	JPXK00000000	163	4,879,936	4,477	United States
2011EL-1497-2	O6:NM	*eltA, st1b, lngB, prfB*	JPUU00000000	319	4,965,333	4,565	Cruise ship
2011EL-1640-5	O6:H16	*eltA, st1b, lngB, prfB*	JPXN00000000	309	4,872,979	4,470	United States
2011EL-1251-4	O6:H16	*eltA, st1b, lngB, prfB*	JPXM00000000	239	4,881,768	4,449	Cruise ship
F6097	O6:H16	*eltA, st1b, lngB, prfB*	JPXJ00000000	292	5,185,255	4,801	United States
F5995	O6:H16	*eltA, st1b, lngB, prfB*	JPXL00000000	271	5,169,984	4,784	United States
							

a Strains 2013EL-1319 and 2013EL-1320 are from the same cruise ship outbreak.

b Strains 2011EL-1369-1 and 2011EL-1370-2 are from the same outbreak in United States.

ETEC genomic DNA was extracted using the DNeasy blood and tissue kit (Qiagen, MD), according to the manufacturer’s protocol. A library preparation for whole-genome sequencing was generated using the Nextera DNA sample preparation kit (Illumina, Inc., CA) from 10 isolates, with a starting concentration of 10 ng/µl. Whole-genome sequencing was performed using MiSeq (Illumina, CA), according to the manufacturer’s protocols, to generate 2 × 150-bp reads. The raw reads were trimmed and assembled in CLC Genomics Workbench 7.0 by *de novo* assembly. The sequences were annotated with the NCBI Prokaryotic Genome Automation Pipeline (http://www.ncbi.nlm.nih.gov/genome/annotation_prok/).

The average size of the ETEC genomes in this study is 4.88 Mb, with 4.7 Mb being the smallest genome size (isolate 2013EL-1319, Table 1) and 5.18 Mb the largest genome size (isolate F6097, Table 1). On average, 4,094 coding sequences were identified in the 10 ETEC genomes (Table 1). BLAST and virulence finder software tools identified the classical enterotoxin genes: LT and ST1b, which were experimentally confirmed by real-time PCR assays (Table 1). The *ingA* gene, which encodes longus type IV pilus, a classical ETEC colonization surface antigen, was found in five ETEC genomes, and the *prfB* gene, which encodes a P-related fimbriae regulatory gene, was found in all 10 ETEC genomes (Table 1).

### Nucleotide sequence accession numbers.

The annotated draft whole-genome sequences of ETEC are deposited in DDBJ/ENA/GenBank. These accession numbers (Table 1) are JPIF00000000, JPUD00000000, JPUS00000000, JPUT00000000, JPXK00000000, JPUU00000000, JPXN00000000, JPXM00000000, JPXJ00000000, and JPXL00000000. A detailed report on further analyses of some or all of the draft genome sequences will be released in a future publication.
