# Erratum for Pattabiraman and Bopp, Draft Whole-Genome Sequences of 10 Serogroup O6 Enterotoxigenic *Escherichia coli* Strains

**DOI:** 10.1128/genomeA.00394-16

**Published:** 2016-05-05

**Authors:** Vaishnavi Pattabiraman, Cheryl A. Bopp

**Affiliations:** Centers for Disease Control and Prevention, Atlanta, Georgia, USA

## ERRATUM

Volume 2, no. 6, e01274-14, 2014. Page 1: The first sentence of the third paragraph should read as follows. “The average size of the ETEC genomes in this study was 4.88 Mb; 2013EL-1320 (Table 1) had the smallest genome, at 4.71 Mb, and F6097 (Table 1) had the largest genome, at 5.18 Mb.”

Page 1: In the last column of Table 1, the country/location of outbreak for ETEC isolates 2013EL-1319 and 2013EL-1320 should read “Haiti.”

